# An Innovative Framework for Sustainable Development in Healthcare: The Human Rights Assessment

**DOI:** 10.3390/ijerph19042222

**Published:** 2022-02-16

**Authors:** Flaviu Moldovan, Petruta Blaga, Liviu Moldovan, Tiberiu Bataga

**Affiliations:** 1 IOSUD Doctoral School, “George Emil Palade” University of Medicine, Pharmacy, Science, and Technology of Targu Mures, 540142 Targu Mures, Romania; 2 Faculty of Economics and Law, “George Emil Palade” University of Medicine, Pharmacy, Science, and Technology of Targu Mures, 540142 Targu Mures, Romania; petruta.blaga@umfst.ro; 3 Faculty of Engineering and Information Technology, “George Emil Palade” University of Medicine, Pharmacy, Science, and Technology of Targu Mures, 540142 Targu Mures, Romania; liviu.moldovan@umfst.ro; 4 Department of Orthopedics—Traumatology, “George Emil Palade” University of Medicine, Pharmacy, Science, and Technology of Targu Mures, 540142 Targu Mures, Romania; tbataga@gmail.com

**Keywords:** healthcare, sustainable development, human rights, assessment, reference framework, facility

## Abstract

Healthcare providers are investing considerable resources for the development of quality management systems in hospitals. Contrary to these efforts, the number of tools that allow the evaluation of implementation efforts and the results of quality, security and sustainable development is quite limited. The purpose of the study is to develop a reference framework for quality and sustainable development in healthcare, Sanitary-Quality (San-Q) at the micro system level, which is compatible with applicable national and international standards in the field. The research method consisted of the study of literature, identification and analysis of good sustainability practices in healthcare, which allowed identification of the areas of the new San-Q framework: quality, economic, environmental, social, institutional and healthcare. These areas are incorporated into the core topics of social responsibility mentioned by ISO26000. A total of 57 indicators have been defined that make up the new reference framework. The evaluation format of the indicators is innovative through a couple of values: completion degree–significance. In the experimental part of the research, a pilot implementation of the San-Q framework at an emergency hospital was performed, the results recorded in terms of responsibility for human rights being presented. The conclusions of the study reveal the innovative aspects of the framework that facilitate the development of a sustainability strategy promoted through performance indicators, the results obtained after evaluation being useful in establishing a reference level of sustainability but also in developing sustainability policies.

## 1. Introduction

Sustainability is defined by the quality of an anthropogenic activity to be carried out without exhausting the available resources and without destroying the environment, and thus without compromising the possibilities of satisfying the needs of the next generations [1]. While quality is defined by the ability to satisfy good execution for activity requirements, continuation of this ability over time is the sustainability which refers to the organizational long-term health. Human rights consist of the whole set of basic rights to which all people are entitled, taking into account two broad categories of human rights, civil and political, which in the context of healthcare are directly related to the promotion of equal opportunities, access to healthcare and also promotion of sustainable and responsible behavior. A sustainable environment is essential for the full observance of a wide range of human rights, including the right to life and healthcare.

In this context, the term “sustainable development” is often used, according to which the needs of current generations are met without depleting available resources, without destroying the environment and without endangering the capacity of future generations to secure the resources they need, which means a good quality of life supporting the human rights for current and future generations [2].

One of the stated objectives of community health policy is to protect citizens from threats, to maintain their health and to support sustainability. New technologies have the capacity to revolutionize healthcare systems, to contribute to their sustainability in the future [3]. E-health, genomics and biotechnology [4,5] can improve disease prevention, treatment management and support the transition from hospitalization to primary care and prevention.

Healthcare providers are investing considerable resources for the development of quality management systems in hospitals. Contrary to these efforts, the number of tools that allow the evaluation of implementation efforts and the results of quality, security and sustainable development are quite limited [6].

In the project “Deeping our understanding of quality improvement in Europe” (DUQuE) [7], organizational culture was associated with some structural features without establishing a direct relationship with the implementation of quality management in healthcare facilities. Moreover, ref. [8] developed seven methods for measuring quality management at the hospital level, which reveals a more comprehensive picture of the quality management implementation degree in hospitals, at different levels and in different hospital departments.

Unlike the usual commercial activities, which are essentially amoral, healthcare is an activity with an intrinsic moral purpose, in which the range of corporate misconduct is limited, making health organizations more difficult to sanction [9]. The concept of health responsibility is a characteristic element of public health policies but also of public discourses on health. Snelling [10] points out that the utilization of this concept is defective, conceptually inconsistent, and therefore needs to be remedied.

G8 commitments, but also global initiatives to strengthen health systems, have paid more attention to the factors that affect health system performance. The results of research by Gruskin et al. [11] indicate the potential benefits of including human rights in these approaches, which would have the effect of increasing participation and involvement of clients in health systems, would facilitate the expansion of the concept of equity, through better awareness of policies and laws beyond current regulations and, through this, would help strengthen accountability mechanisms.

La Rosa et al. [12] believe that new approaches to social responsibility and health need to be developed to ensure that scientific and technological progress contributes to justice, equity and the interests of humanity. Houtepen and Meulen [13] developed the concept of reflective solidarity which, together with the concept of responsibility of healthcare institutions, political decisionmakers and citizens as recipients of healthcare, includes elements of social justice.

In this context, a causal hypothesis of the research is formulated, according to which the employment of reference frameworks is efficient in the sustainable development of healthcare facilities at the micro system level, promotes sustainable and responsible behavior and, through access to healthcare and equal opportunities, provides respect for human rights.

The general objective of this research is to define and develop a reference framework for sustainable development in healthcare, at a micro system level, which is compatible with the applicable national and international standards in the field.

The specific objectives of the research are:

1. To identify the core activities of a framework for sustainable development in healthcare;
2. To select the best practices from healthcare systems, which are reported worldwide in meta studies and which are related to the implementation of quality management systems and sustainable development activities;
3. To design the indicators that make up the framework for sustainable development in healthcare;
4. To develop a method for indicator evaluation;
5. To validate in practice the new innovative framework for sustainable development.

## 2. Materials and Methods

The scientific research in this paper is exploratory and has been used as research methodology:

1. Design of the areas of the new framework according to the quality cycle by employment of sustainability concepts, legal norms in the medical field, requirements of the social responsibility standard and other similar existing frameworks;
2. Identification of the successful sustainable medical practices presented in the scientific literature that fall within the areas of the new framework and which can be used to describe indicators;
3. Conception of the framework indicator matrix;
4. Design of the indicators content and the method for evaluation by using the relevant practices from the medical literature;
5. Pilot implementation of the framework aimed at validating in practice its content and the associated methodological approach.

### 2.1. Areas of the New Reference Framework

In the research, it was necessary in the first stage to establish the areas that make up the new reference framework.

The fields of the new reference framework were established by exploring the medical scientific literature and by collecting the most relevant ideas, which allow a causal and significantly positive relationship between a good organization and sustainability [14].

At this stage, in view of Isaksson’s conclusions [15], it was considered the symbiosis of practical interest between quality management and sustainability, which need to be further explored for a healthcare unit. Zdravkovic and Radukic [16] point out that, in addition to the 3 established areas of sustainability, social, environmental and economic, it is advisable to incorporate the institutional dimension which can manage sustainable development, in the interest of present and future generations.

After analyzing the 4 conceptual components of the frameworks developed so far and presented in the literature, we considered medical services as the 5th core area of a healthcare facility.

**Figure 1:**
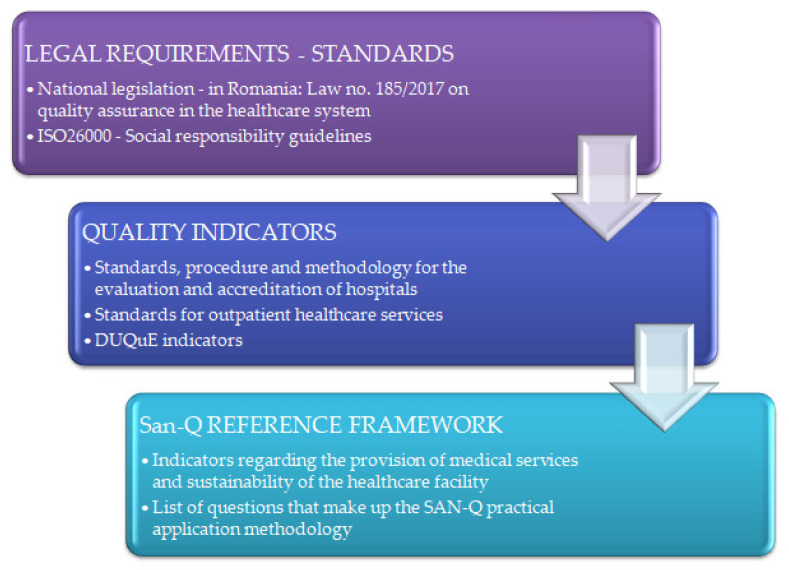
Conceptual model for the San-Q reference framework development.

The conceptual model for the development of the San-Q framework is presented in Figure 1, which incorporates the legislation and medical standards, as well as the indicators from the existing reference frameworks [17].

Based on the global requirements, standards, procedure and methodology for the evaluation and accreditation of hospitals [18], as well as the standards of the National Authority for Quality Management in Health (ANMCS) for outpatient health services [19], but also regional specificity, the San-Q framework has been developed. It takes into account that the healthcare system can and should play a key role in promoting community coherence and should also seek environmental responsibility and financial stability.

**Figure 2:**
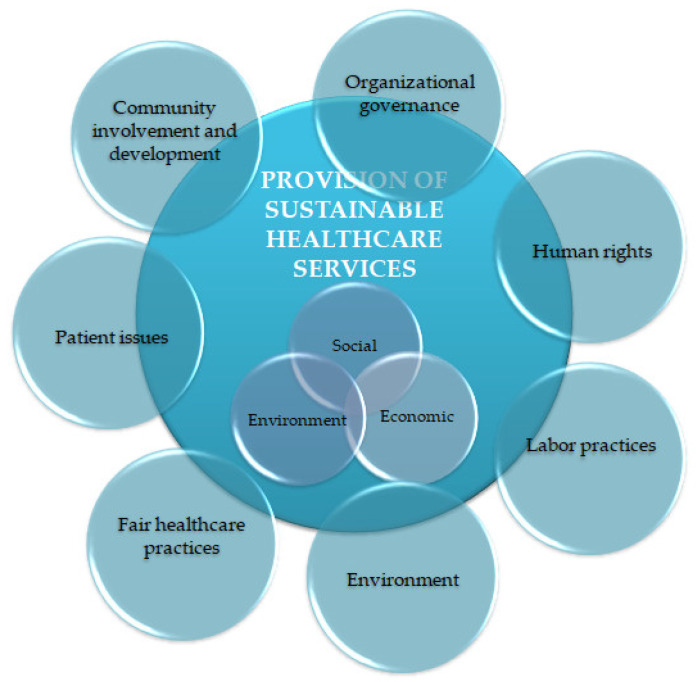
The pillars of sustainable development and the social responsibility core topics according to ISO 26000.

For this reason, the San-Q reference framework is structured on the 3 dimensions of sustainable development, social, environmental and economic, to which the quality of institutional governance and healthcare are added, that are integrated in the seven core subjects of the standard ISO26000—Social responsibility guidelines [20], governance, human right, labor, environment, business practices, consumer and community, adapted to the context of healthcare provision (Figure 2).

With this support, the San-Q reference framework was structured on the 4 main phases of the quality cycle, Planning—Implementation—Evaluation—Review (PIER), that were adapted to the healthcare design of medical services provision, medical services provision, medical services evaluation and continuous improvement.

In the Planning phase, the San-Q reference framework includes the design of medical services provision that covers all aspects related to the definition of healthcare provision in the healthcare facilities. The Implementation phase consists of providing healthcare services that represent the process of treating patients. In the Evaluation phase, the San-Q reference framework includes the evaluation of healthcare services that comprises all aspects related to the evaluation of patient satisfaction, the effectiveness of treatment and the evaluation of medical staff satisfaction. The Review phase takes place after the provision of healthcare services and their evaluation, by conducting self-assessments and redesigning healthcare services, thus ensuring continuous improvement.

In order to establish the indicators that make up the San-Q reference framework, each of the four phases of the quality cycle were divided into two basic activities: P.A. Accreditation of healthcare services, P.B. Patient-centered care interventions design, I.A. Healthcare provision, I.B. Transfer assurance, E.A. Local opinion leaders’ evaluation and involvement, E.B. Satisfaction assessment, R.A. Self-assessment and R.B. Healthcare services innovation.

**Figure 3:**
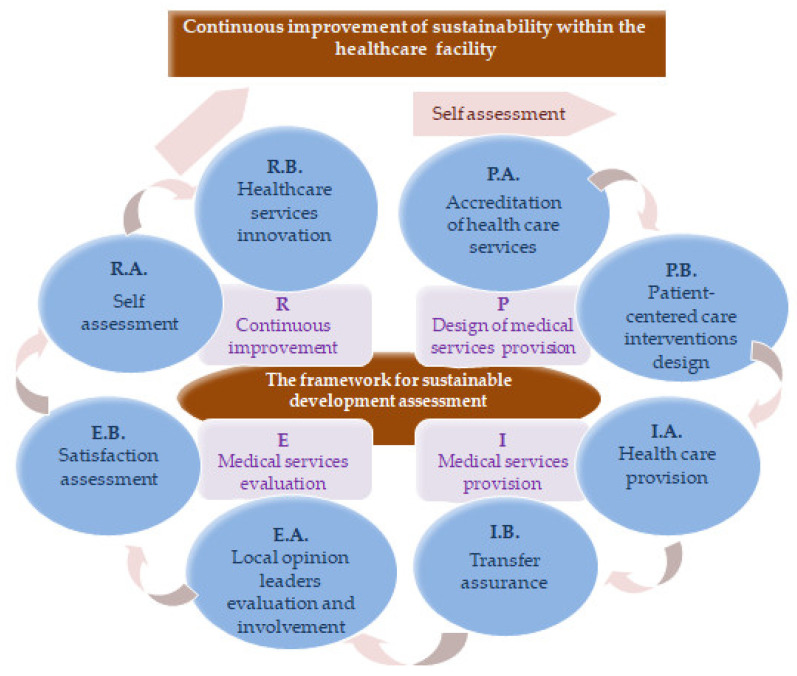
The basic activities of the continuous improvement cycle for the sustainable development of the healthcare facility.

The core activities of the continuous improvement cycle for the sustainable development of the healthcare facility are represented in Figure 3.

### 2.2. Sustainable Medical Practices Presented in the Scientific Literature

In the second stage of the research, in order to design the performance indicators that make up the new reference framework for sustainable development, we conducted a qualitative study of the scientific literature, mainly in the PubMed database. First, we analyzed the healthcare facilities that are considered representative of medical performance, having different levels of human capital and forms of public/private ownership, from which we deduced the most relevant sustainability practices that are confirmed in meta studies.

The relevant approaches regarding quality and sustainability were searched by keywords and collected, after which a comparative analysis was performed and the practices of interest for this research were extracted.

We have explored the practices for quality and sustainable development, and we have found diverse paths that can support healthcare facilities to find a common way for sustainability implementation. The key issues collected and used in the methodology are:

1. Vision/mission/objectives related to quality and sustainability assessment;
2. Previous experience of the healthcare facility;
3. Institutional context and key issues on the transition from sustainability assessment to sustainable development;
4. Key success factors.

The specialized literature exploration allowed the identification of the successful practices, designed and verified in practice, which we present below, grouped on the basic activities of the quality cycle.

#### 2.2.1. P. Design of Medical Services Provision

##### P.A. Accreditation of Healthcare Services

The analysis of the quality assurance processes evolution within the hospital is marked by two main stages, namely the professional administrative stage and the organizational stage [21]. Alaraki’s research findings [22] indicate a direct correlation between hospital performance and the 8 total quality management practices: leadership, staff involvement, information processing, continuous training, customer orientation, continuous improvement, process approach and provider management. Hospital accreditation may orient these organizations to quality management but does not appear to improve overall patient satisfaction [23].

Some studies compare the quality of care in accredited and non-accredited hospitals for different medical specialties and conclude that the accreditation of the hospital as well as of some of its specialties, such as acute myocardial infarction [24,25], traumatology [24], outpatient surgery and infection control [26], have the effect of improving medical processes, healthcare services and the organization and operation of the hospital.

Carotid duplex ultrasound scan results reveal significant overestimation by unaccredited laboratories of carotid artery stenosis [25]. Lack of accreditation has been independently associated with suboptimal management of sleep medications [25]. It has been statistically proven that the survival rate of patients with severe trauma who are treated in accredited hospitals is higher than the survival rate in non-accredited hospitals [25]. There were lower mortality rates in patients who were treated in accredited hospitals [27].

A meta-analysis of 78 articles concludes that accreditation is increasingly used as a tool which facilitates the quality improvement of medical services in low- and middle-income countries, which have established national hospital accreditation programs tailored to their national contexts [28].

##### P.B. Patient-Centered Care Interventions Design

The effectiveness of quality patient-centered interventions was investigated in randomized controlled trials and controlled clinical trials. The implementation of patient-centered home healthcare has been strongly associated with important outcomes for both patients and providers [29].

According to the study of Groene et al. [30], there is a lack of evidence that patients’ involvement or their representatives in hospital quality management leads to the establishment or implementation of strategies and procedures to facilitate patient-centered care, but the lack of evidence should not be construed as evidence for lack of effect [30].

The effectiveness of training interventions has been demonstrated, and the distribution of informative material has positive effects on the medical consultation processes regarding increased detection of psychological suffering, increase in the medical consultations proportion in which all the patient’s health problems were analyzed and improvement of the patient’s perception of the disease-specific information [31].

Moreover, mixed effects on medical consultation processes as determined by medical staff were identified regarding patient-centered communication behavior, empathy skills, use of various data collection skills as well as co-decision by patient involvement [32].

Depending on the direct needs of the patient, the medical staff prescribes the most appropriate patient-centered approach, ensuring that it meets the three essential requirements: partnership, communication and health promotion [33]. However, the results of the study by Groene et al. [30] show that there is a need for better integration of patient-centered care in quality management, and the motivation and impact of patient involvement in service design and evaluation are assessed in a nuanced way.

#### 2.2.2. I. Medical Services Provision

##### I.A. Healthcare Provision

The effects of the computer applications developed for implementation of quality management in hospitals are the efficiency of the quality management processes by reducing the number of documents, the information to be completed and the tasks but also the information errors [34].

Jarvis et al. [35] assessed the impact of advanced electronic health records use on the quality of hospital services and patient satisfaction. The results suggest that the most advanced electronic health records have the greatest benefit in improving the quality of the clinical care process, without having a negative impact on the patient experience.

The investigation of process data in the quality management system is useful for estimating the results of the measures applied in healthcare, along with the continuous evaluation which is essential [36].

Numerous studies, mostly controlled and randomized, have evaluated computer systems that facilitate clinical decisions and provide strong arguments for their effectiveness [37]. Studies have shown that computerized clinical decision support systems, both commercially developed and locally developed, lead to:

1. Substantial increases in the identification of the adverse events and adverse drug rates [38];
2. Consistent improvement of preventive care services [39];
3. Adequacy of treatment and therapy ordered by providers in terms of conducting clinical trials [40] and reduction in hospitalization costs [41,42];
4. Improvement of some morbidity outcomes, but studies have limited ability to detect significant clinical differences in mortality [42].

Continuing education may involve care providers in the continuous improvement of quality, but the dissemination of knowledge from trained staff to other staff remains limited [43]. An effective medical education requires a combination of teaching methods: online courses (self-study), face-to-face courses (traditional method) and a database of materials [44].

Studies on single interventions show that median improvements in healthcare can be achieved with the support of the educational materials [45] and reminders [46] but also audit and feedback [47].

Studies on multiple interventions combining educational materials, educational meetings, reminders, auditing and feedback conclude that multiple interventions are not more effective than individual interventions and the increase is not proportional to the number of interventions [48].

Overall, all studies consider that there is little evidence to support the link between organizational culture and health performance [49], and the articulation of this relationship is proved to be difficult [50]. Current available evidence does not identify effective and generalized strategies for changing organizational culture [51], although it is appreciated that investing in strategies to encourage a high-performance organizational culture can assist hospitals in their efforts to improve clinical outcomes [52].

##### I.B. Transfer Assurance

A number of studies have evaluated the effectiveness of interventions aimed at improving hospital transfers. The transfer of technological solutions that are used can lead to the prevention and reduction in adverse events and a better transfer quality satisfaction [53].

The study of healthcare reveals that supplementing verbal communication with a written environment leads to improved information retention [53]. “White Papers” and “Clinical Consensus Statements” [54] allow an efficient, uninterrupted verbal exchange. The allocation of interview duration is focused on the sick patients and on the actions needed to be taken, but the content needs to be continuously updated in order to ensure the communication of the latest clinical information [53].

Although education to improve transfer has not been shown to improve patient outcomes, it does improve the attitudes, knowledge and skills of healthcare professionals in the workplace [55].

#### 2.2.3. E. Medical Services Evaluation

##### E.A. Local Opinion Leaders’ Evaluation and Involvement

Initial medical education and continuous medical education are not sufficient to change the behavior of doctors and health professionals. Healthcare professionals can appeal on opinion leaders to positively influence the clinical environment and their colleagues but without formalizing their role so as not to dilute the professional influence [56].

The identification of opinion leaders based on personal characteristics and interpersonal networks is described by Holliday et al. [57]. There is some evidence that the time spent and the work of leaders can influence the quality and safety of clinical outcomes, processes and performance [58].

Flodgren et al. [59], who looked at medical practices in 337 hospitals, concluded that evidence-based medicine can be successfully promoted by opinion leaders, individually or in combination with other interventions, without concluding on how to optimize opinion leaders’ interventions. Although evidence-based practice can be successfully promoted by local opinion leaders, there is no certainty that this widely applied practice is effective [60].

##### E.B. Satisfaction Assessment

Patient satisfaction is considered an indicator that interferes with the effectiveness of interventions. There is evidence from healthcare professionals that patients with a higher level of satisfaction recover faster [61].

It has been found that patient satisfaction increases with the communication skills of the medical staff, and for this reason, healthcare providers must identify the components of the communication that need improvement and then to improve the skills of the medical staff which will increase the level of service provided to patients [62].

The success of group cooperation and coordination is influenced by social capital, and studies show that hospitals with well-developed quality management systems have a higher degree of social capital [63]. By improving social capital, the satisfaction of nurses in the workplace could be improved and lead to a better quality of patient care [64].

#### 2.2.4. R. Continuous Improvement

##### R.A. Self-Assessment

An attractive alternative to the strategy of implementing the quality management system at the level of the entire hospital is the continuous improvement of the quality within the hospital departments, but this option must be validated by successive long-term evaluations [65].

A number of studies based on experimental or quasi-experimental design have investigated in detail the effectiveness of audit and feedback and have shown small and moderate but systematic effects on the effectiveness of professional development [48]. In situations where basic performance is low, the effectiveness of feedback increases if it is repeatedly provided by a colleague or supervisor, verbally or documented [66], and when it shows both explicit measures as well as an action plan [67].

##### R.B. Healthcare Services Innovation

A limited number of individual studies have evaluated the effectiveness of Lean and Six Sigma methods, which have reported improved healthcare in operating rooms, emergency departments and reduced waiting times for patients [68]. Infection control improvement and the increased proportion of non-cardiac patients receiving antibiotics within one hour before surgery has also been reported [69].

In the case of operated patients, it has been found that the Lean and Six Sigma methods have a high potential to improve the clinical condition in terms of optimizing outpatient efficiency, improving operating room efficiency, reducing surgical complications, reducing in-department medical incidents, reducing mortality and limiting unnecessary costs and duration of hospitalization [70], which can be reduced by about three days for surgical patients [69].

Protocol compliance and patient safety improvement are the benefits suggested by studies that have evaluated the effectiveness of patient safety checklists [71].

In the scientific literature, research is available on the effectiveness of incident reporting, which results in reduced mortality and improved patient survival at 1 year, decreased rate of adverse drug events, improved communication between healthcare professionals about patient safety, improved compliance with work processes and patient care monitoring [72].

In order to increase the efficiency of safety feedback, the cycle needs to be closed, and the sequence of reporting, analysis and investigation actions must be completed with corrective actions that must be applied in a timely manner and that effectively address the vulnerable aspects of the working systems [73].

Studies show that educational visits/meetings alone or when combined with other interventions can be effective in improving healthcare processes. Benefits are usually reduced to limited, but potentially important for compliance with the desired practice and prescribed behavior [74]. Changing complex behaviors cannot be achieved with the support of educational meetings alone. The effectiveness of educational meetings can be increased through participatory growth strategies, which employ mixed interactive and didactic formats and which are perceived as serious because they are focused on results [2].

### 2.3. Indicator Matrix of the San-Q Sustainable Development Framework

In the third stage of the research, in order to establish the list of indicators of the San-Q reference framework, each basic activity in the quality cycle (Table 1, PIER lines) was linked to the main basic topics of social responsibility (Table 1, columns 1–7).

Based on the correspondences established between the basic activities of the quality cycle and the basic topics of social responsibility, as well as the sustainable healthcare activities, we designed the indicators matrix of the San-Q sustainable development framework, which contains 57 indicators, as shown in Table 1.

The design of the 57 specific indicators, which make up the San-Q reference framework, required a detailed description of them, accompanied by guiding questions for the evaluation, as well as the elaboration of the method to quantify the degree of fulfillment. All this facilitates the process of self-assessment of healthcare facilities in terms of implementing a sustainable model of continuous quality improvement in the provision of healthcare.

### 2.4. Description and Evaluation Grids of the Human Rights Indicators

In order to illustrate the way in which indicators are defined and for reasons of space constraint, this paragraph sets out the indicators that make up the field of human rights.

Human rights cover the whole set of basic rights to which all people are entitled, taking into account the broad categories of human rights: civil, political, economic, social and cultural rights. In the context of healthcare, they are directly linked to the promotion of equal opportunities, access to healthcare, the promotion of sustainable and responsible behavior and citizenship, respect for human beings and differences, and so on.

In this research, a self-assessment tool was designed to implement the innovative framework for sustainable assessment of healthcare facilities.

The principle of the instrument is to answer the questions taken/derived from the San-Q sustainable development framework and to evaluate these answers. As the questions characterize the indicators of the San-Q framework, the indicators are assessed by evaluating the answers to the questions.

The numerical evaluation of the indicators was chosen because it allows the establishment of benchmarks [75], the implementation progress monitoring, as well as monitoring long-term sustainability trends [76]. The information provided allows for an X-ray of the current situation and relevant decisions for the future [77].

In this paper, a new modality for numerical evaluation of the San-Q framework indicators is proposed. The values used are of an information pair composed of the realization of the indicator–the significance of the indicator [78]. Both categories of information are classified by numerical values from 0 to 5.

The format of the indicators’ significance is presented in Table 2. The significance categories are: 0—Not applicable, 1—Insignificant, 2—Reduced significance, 3—Significant, 4—Very significant, 5—High significance.

Completion degree of the indicators is evaluated by numerical values from 0 to 5, corresponding to the fulfillment categories which are: 0—Not applicable, 1—Weak, 2—Satisfactorily, 3—Good, 4—Very good, 5—Excellent.

Table 3, Table 4, Table 5, Table 6, Table 7, Table 8, Table 9, Table 10, Table 11, Table 12, Table 13, Table 14, Table 15, Table 16, Table 17 and Table 18 show the evaluation grids of the indicators that make up the cycle of continuous improvement of human rights in the healthcare facility.

**Figure 4:**
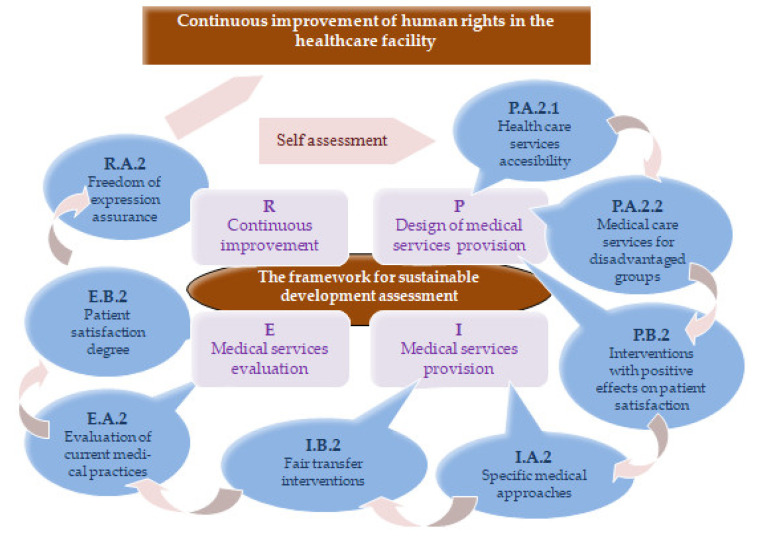
The cycle of continuous improvement of human rights in the healthcare facility.

The employment of the indicators presented in Table 3, Table 4, Table 5, Table 6, Table 7, Table 8, Table 9, Table 10, Table 11, Table 12, Table 13, Table 14, Table 15, Table 16, Table 17 and Table 18 allows the evaluation of human rights in the healthcare facility in the four stages of the quality cycle (Figure 4): Planning—P.A.2.1 Healthcare services accessibility, P.A.2.2 Medical care services for disadvantaged groups, P.B.2 Interventions with positive effects on patient satisfaction; Implementation—I.A.2 Specific medical approaches, I.B.2 Fair transfer interventions; Evaluation—E.A.2. Evaluation of current medical practices, E.B.2 Patient satisfaction degree; Review—R.A.2 Freedom of expression assurance.

## 3. Results

In the experimental part of the research, a pilot implementation of the framework for sustainability assessment was performed at the Emergency County Hospital Targu Mures (ECHM) [79]. It is a public healthcare facility that is in the Ministry of Health suborder, classified in category I—very high level of competence—which has a complex structure of specialties of which 42.57% are surgical and 57.43% are physician specialties.

The pilot implementation aimed to validate the presented methodological approach and to provide a good example for interested users. This was conducted during one week by a group of four evaluators with different responsibilities in the field of healthcare and quality management, who are involved in the implementation of the quality management system, in different jobs: head of department, doctor, responsible for quality assurance and nurse.

The evaluation was performed for each of the three dimensions of sustainability that are integrated in the seven social responsibility core subjects, by ranking the values of the couple (completion—significance of the indicators) from 0 to 5. This paper, for reasons of space, presents the results recorded in terms of responsibility for human rights, for which the assessments was performed based on the evaluation of the indicators. The findings are presented below.

P.A.2.1 Healthcare services accessibility—ECHM, together with the structures within the Emergency Institute for Cardiovascular Diseases and Transplantation Targu Mures, fulfills the role of regional emergency hospital with IA competence level for the Center Region. The characteristics of the population in the addressability area of the hospital are identified. The addressability of the hospital reflects its ability to provide medical services for which there is an increased need and demand, which is associated with the offer of unique and high-quality healthcare services.

The analysis of morbidity data shows that the need for medical services of the population is covered by the hospital at the level of major classes of diseases, except for infectious diseases, respiratory diseases and cardiovascular diseases. The incidence and prevalence of cardiovascular disease and digestive disorders would justify an increase in the number of beds.

P.A.2.2 Medical care services for disadvantaged groups—ECHM runs regional programs for the prevention, early diagnosis and early treatment of major pathologies (e.g., cervical cancer for 170,200 women in the Center Region), which aims to promote the social inclusion of vulnerable groups by increasing access to healthcare and social services of general interest.

P.B.2 Interventions with positive effects on patient satisfaction—The doctors in charge of the departments and the doctors responsible for quality management in collaboration with the employees of the medical quality management service draw up the general report on the analysis of the diagnostic and treatment protocols. Together with the results of the evaluation and the proposed recommendations, this is sent to the Hospital’s Steering Committee in order to formulate improvement measures.

I.A.2 Specific medical approaches—There is an integrated team medical approach for the purpose of multidisciplinary care. The patient’s treatment plan is a result of collaborative processes between physicians of different specialties. Taking into account the wishes of the patient and the best medical treatments available, it is prescribed to the patients’ specific treatments. The multidisciplinary approach has the effect of increasing the quality of patient’s life, decreased mortality and morbidity.

I.B.2 Fair transfer interventions—Inter clinical transfer protocols are applied for critical patients to healthcare facilities with a higher level of competence and endowment, which can provide complex and complete care for a particular pathology. During the patient’s transfer, the service provides medical supplies and uncollected medicines.

E.A.2. Evaluation of current medical practices—The evaluation of the medical work protocols efficiency and effectiveness formalized on the medical activities in each section/compartment of the hospital is performed based on the indicators established in the protocol. The system procedure on the methodology of diagnostic evaluation and treatment protocols is also used by the head of quality management nominated within the ward and the medical director. They are valued as local opinion leaders with professional influences on the ward staff. They draw up a report on the analysis of diagnostic and treatment protocols in which the evaluation results are presented and recommendations are made in order to improve medical services.

E.B.2 Patient satisfaction degree—In order to evaluate the medical services provided during the hospitalization period, in 2020, the quality management service of the health services processed 1519 questionnaires which were applied to the patients, through which the attitude of the staff (68.86%—very satisfied, 24.62—satisfied, 6.25%—no answer, 0.26%—dissatisfied), quality of accommodation conditions—lounge—endowment, facilities (51.28%—very satisfied, 41.01—satisfied, 6.25%—no answer, 1.45%—dissatisfied), general degree of satisfaction (66.36%—very satisfied, 30.81—satisfied, 2.63%—no answer, 0.20%—dissatisfied) [79], etc., were evaluated.

Moreover, 6839 questionnaires collected from inpatients were processed, which monitored the patient satisfaction degree on the distribution of food (49.18%—very satisfied, 41.01%—satisfied, 7.18%—did not answer, 2.63%—dissatisfied), quality of meals served (54.05%—very satisfied, 37.72%—satisfied, 6.45%—did not answer, 1.78%—dissatisfied) [79]. Patient testimonials were collected and published online.

The analysis of the answers and the interpretation of the results show that the biggest dissatisfaction of the patients is related to the fact that they have to change their clothes in the hospital ward and the fact that they have to buy medicines for hospitalization. By taking appropriate measures to ensure privacy for changing clothes and provision of free medicines, an immediate increase in the patient’s satisfaction degree could be achieved.

R.A.2 Freedom of expression assurance—A patient feedback mechanism is implemented. It consists of the analysis of the medical services offered, the degree of observance of the obligations and rights by medical staff and by patients. The evaluation questionnaire and the online referral form are made available to patients, and the submitted documents are analyzed by the Ethics Council.

The values of human rights indicators are presented in the self-assessment tool (Table 19).

**Figure 5:**
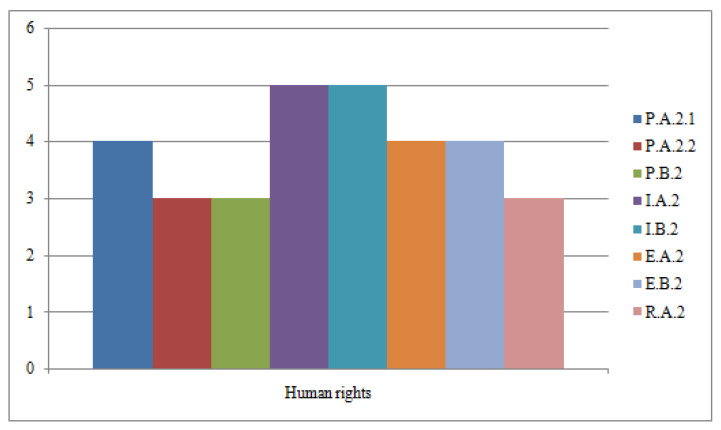
Degree of fulfillment for human rights responsibility indicators.

The completion degree of human rights responsibility indicators, on a scale of 1 to 5, is shown graphically in Figure 5. Three indicators, namely PA2.2 Medical care services for disadvantaged groups, PB2 Interventions with positive effects on patient satisfaction and RA2 Freedom of expression assurance, have the completion degree 3, the lowest in this group, while the highest completion degree 5 is registered for the two indicators IA2 Specific medical approaches and IB2 Fair transfer interventions.

**Figure 6:**
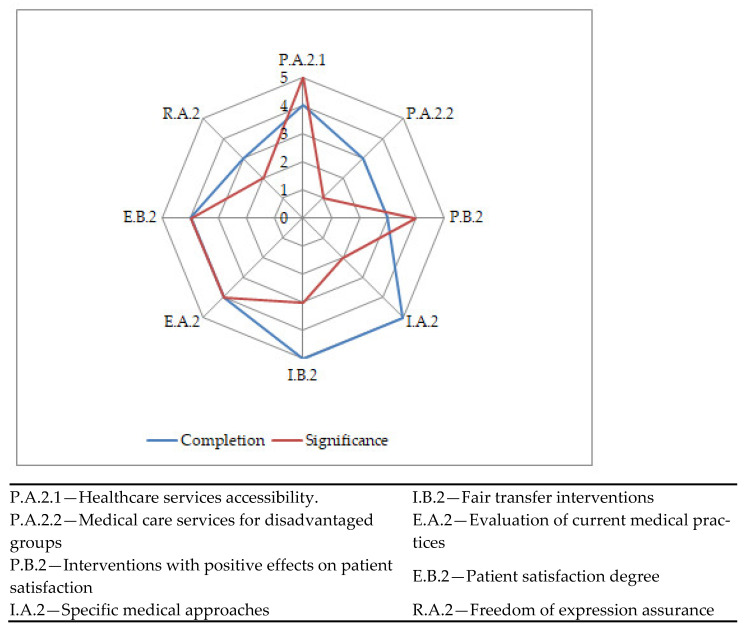
Human rights sustainability assessment chart.

The results for the human rights performance indicators are shown in Figure 6. This is the assessment chart represented in terms of completion degree and significance.

The overall quality indicator for human rights responsibility (IGQ_HR_) is obtained by summing the values of the quality indicators for all eight indicators evaluated (see Table 11):

(1) $${\mathrm{IGQ}}_{\mathrm{HR}}=\overset{8}{\underset{i=1}{\sum }}{\mathrm{IQ}}_{i}=\overset{8}{\underset{i=1}{\sum }}S_{i}\cdot G_{i}=98$$

The maximum value of the global quality indicator for human rights responsibility (IGQmax_HR_) is obtained if the degree of completion of each indicator is maximum and it is evaluated with the value 5, in which case it is calculated with the formula:

(2) $${\mathrm{IGQmax}}_{\mathrm{HR}}=5\cdot \overset{8}{\underset{i=1}{\sum }}S_{i}=5\cdot 25=125$$

The overall quality level of human rights accountability (LGQ_HR_) is calculated as the ratio between the global quality indicator for human rights responsibility (IGQ_HR_) and the maximum value of the global quality indicator for human rights responsibility (IGQmax_HR_), multiplied by 100, in order to be expressed as a percentage:

(3) $${\mathrm{LGQ}}_{\mathrm{HR}}=\frac{{\mathrm{IGQ}}_{\mathrm{HR}}}{{\mathrm{IGQmax}}_{\mathrm{HR}}}\cdot 100=\frac{98}{125}\cdot 100=78.40\%$$

The overall level of quality of human rights accountability provides an overview of the overall state of the organization in relation to the human rights requirements of the San-Q framework.

**Figure 7:**
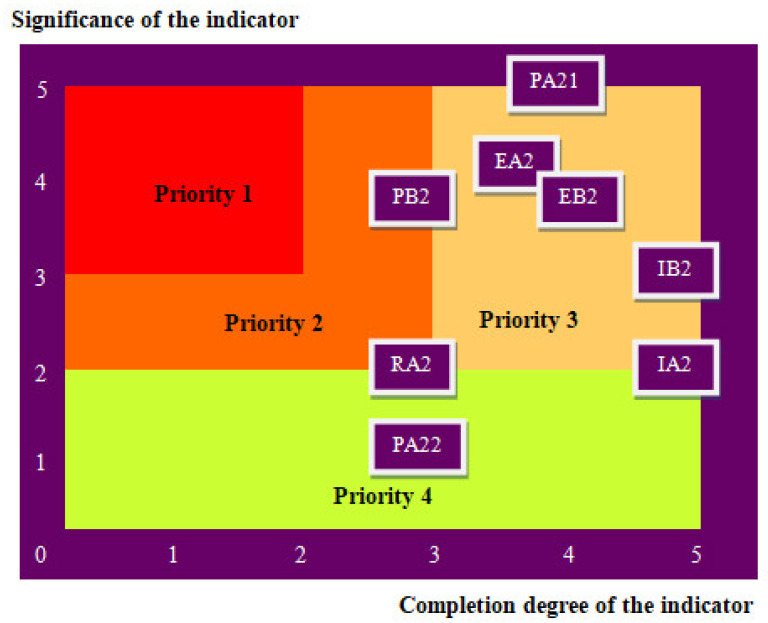
Human rights sustainability assessment diagram.

Human rights outcomes are represented on the sustainability assessment diagram (Figure 7). This is an Eisenhower matrix that provides an overview of human rights sustainability assessment and helps set priorities on a scale of 1—high to 4—low, as well as decision making. The analysis of the diagram shows that the highest priority should be given to the indicators P.B.2—Interventions with positive effects on patient satisfaction and R.A.2—Freedom of expression assurance.

## 4. Discussion

The pilot implementation of the new reference framework for quality and sustainable development in healthcare, Sanitary-Quality (San-Q), has validated in practice the theoretical model at the micro system level. This is an application model that can guide interested users.

Due to the multitude of indicators used in the new framework for sustainable development, the methodological approach requires the participation of several experts from the healthcare facility to work in an evaluation team.

For the success of the evaluation, it was revealed that the professional experience of the evaluators is essential, as well as the way in which they are coordinated by the group leader.

The involvement of a mixed team of evaluators has sometimes led to intense discussions regarding the degree of fulfillment of the indicators but also to the improvement of question content for evaluation and the evaluation grids.

Overall, the expert evaluators were pleased with the participation in this experience, which allowed them to analyze the hospital from another perspective, much more complex, different from their daily professional concerns. They highlighted the positive side of the evaluation by analyzing the aspects that the organization does not evaluate on a daily basis.

The smooth running and objectivity of the evaluation results is conditioned by the availability and accuracy of the data collected from the evaluated department. For this reason, by providing complete information in the planning stage of the audit, the motivation of the participation may increase toward the evaluated requirements.

One suggestion for improvement, collected during the evaluation, was the development of a glossary explaining in more detail some key concepts that might be useful for inexperienced evaluators.

A general remark is that, even if the San-Q framework establishes some correspondence to the evaluation matrices of the DUQuE framework for the evaluation and improvement of quality and safety in European hospitals [80], there are differences between them as well as from the other frameworks used in healthcare, in principle by its objective which promotes sustainable development. Overall, it can be appreciated that the result of the pilot evaluation is in line with other results reported in literature that assess the sustainability of training organizations with the support of reference frameworks [78,81].

We found that hospital accreditation is correlated with quality management practices and improves some medical practices, but not overall patient satisfaction, as reported by Sack et al. [23]. Interventions with positive effects on patient satisfaction need a better integration of patient-centered care as also remarked by Groene et al. [30]. Computer systems and advanced electronic health records use facilitate the clinical decisions of multidisciplinary care teams in specific medical approaches as presented in other studies [35,36]. Reduction in adverse events at ECHM would require an improvement of fair transfer interventions, along with the improvement of the media as reported in research [53,54] and a number of state-of-the-art technical means. Moreover, if the patient satisfaction for the current medical practices is evaluated on a regular basis and a patient feedback mechanism is implemented that ensures the freedom of expression, it may support a faster recovery, as also concluded in [61].

With regard to the results of the human rights assessment at ECHM, the improving measures and resources allocated require the hospital to make efforts to identify new care services for disadvantaged groups, to take measures to increase patient satisfaction through the provision of patient-centered healthcare and the implementation of interventions proposed by local opinion leaders.

The successful activities, designed and verified in practice, which were collected from the scientific literature, allowed the design of performance indicators that are suitable for sustainability assessment of a healthcare facility. In this way, the causal hypothesis of the research is confirmed, according to which the employment of a reference framework is efficient in the sustainable development of a healthcare facility. The sustainable and responsible behavior is promoted, and respect for human rights through access to healthcare and equal opportunities is provided.

A limitation of the study comes from the fact that, in the development of the new framework, research does not cover all the organizational strategies but rather provides an opportunity for managers to reflect on quality and sustainable development approaches organization wide.

Another limitation of the study comes from the validation in practice of the new reference framework that was carried out in an emergency hospital, which is a public unit, with a complex structure of medical specialties. Multiple evaluations are required in healthcare facilities of different sizes and different forms of ownership, which would allow some improvements of the indicator contents so that they take into account a wide range of particular organizational aspects.

Future research directions may consist of the elaboration of a methodology for evaluation with the support of the sustainability framework and a specific assessment electronic tool with interactive links that would help to make it more operational.

## 5. Conclusions

In this paper, a new and innovative framework for sustainable development in healthcare is proposed. It is compatible with hospital accreditation standards and differs from it, as well as other systems, through addressing healthcare facilities that continuously improve the effectiveness of the management system by establishing performance indicators in order to develop and guide toward sustainable development.

The framework based self-assessment supports medical institutions in establishing the level of performance in all areas of the reference framework, identifying opportunities for improvement in the performed activities and deciding on priority measures included in action plans. This is an innovative aspect of the reference framework which supports the development of a high-performance strategy that integrates sustainable development.

The final results deduced from the framework-based evaluation are useful in setting sustainability benchmarks, development of sustainability policies, action plans that include improvement activities with priority implementation that ultimately lead to improving the internal sustainability performance of the organization.

All this will have the effect of increasing the social and economic efficiency of the healthcare facility but also an improved image.

The medical staff, but also the patients of the healthcare facilities that implement the reference framework for sustainable development, will be directed toward sustainability.

## Figures and Tables

**Table 1:** Indicator matrix of the San-Q sustainable development framework.

Indicator Matrix of the San-Q Reference Framework		1. Organizational Governance	2. Human Rights	3. Labor Practices	4. Environment	5. Fair Healthcare Practices	6. Patient Issues	7. Community Involvement and Development
P. Design of medical services provision	P.A. Accreditation of healthcare services	P.A.1. Decision structures and processes	P.A.2.1. Healthcare services accessibility P.A.2.2. Medical care services for disadvantaged groups	P.A.3. Change and professional development promotion	P.A.4. Plan for environmental impact	P.A.5. Attitudes of the profession toward accreditation	P.A.6. Performance information	P.A.7. Community involvement activities
	P.B. Patient-lefted care interventions design	P.B.1. Quality assurance processes design	P.B.2. Interventions with positive effects on patient satisfaction	P.B.3. Quality of patient-lefted medical interventions assurance	P.B.4. Environmental criteria for the selection of materials used in interventions	P.B.5. Effective interventions implementation	P.B.6. Patient self-care design and self-management	P.B.7. Content of the interventions adapted to the community
I. Medical services provision	I.A. Healthcare provision	I.A.1. Computerized support systems for clinical decisions	I.A.2. Specific medical approaches	I.A.3.1. Continuous medical education I.A.3.2. Dissemination and use of clinical practice guidelines	I.A.4.1.Usability of recycled materials I.A.4.2. Recycling of waste	I.A.5. Promotion of the patient safety culture	I.A.6. Critical features for improving the surveillance of patients with chronic conditions	I.A.7.1.Networking and partnership I.A.7.2. Involvement of volunteers and training networks
	I.B. Transfer assurance	I.B.1. Transfer evaluation mechanisms	I.B.2. Fair transfer interventions	I.B.3. Interventions to improve transfers	I.B.4. Environmentally friendly transfer interventions	I.B.5. Features that affect transfer effectiveness	I.B.6. Interventions to reduce problems in outpatients	I.B.7. Involvement and participation of professional associations
E. Medical services evaluation	E.A. Local opinion leaders’ evaluation and involvement	E.A.1. Existence and recognition of local opinion leaders	E.A.2. Evaluation of current medical practices	E.A.3. Improving professional practices	E.A.4. Environmental consumption improvement	E.A.5. Effective work practices	E.A.6. Patient-specific issues management	E.A.7.Local opinion leaders involved in the community
	E.B. Satisfaction assessment	E.B.1. Monitoring mechanisms assignment	E.B.2. Patient satisfaction degree	E.B.3. Satisfaction of medical staff	N/A	N/A	E.B.6. Patient satisfaction degree regarding therapeutic benefits	E.B.7. Satisfaction regarding partnerships
R. Continuous improvement	R.A. Self-assessment	R.A.1. Self-assessment tools	R.A.2. Freedom of expression assurance	R.A.3. Audit and feedback	R.A.4.Waste generation and energy consumption surveillance tools	R.A.5. Feedback to medical staff	R.A.6. Complaints management	R.A.7. Communitarian initiatives
	R.B. Healthcare services innovation	R.B.1. Changes to healthcare services	N/A	R.B.3. Six Sigma and Lean employment in medical organization	R.B.4.Measures applied to the environment	R.B.5. Safety checklists	R.B.6. Incident report	R.B.7. Educational visits

**Table 2:** Significance of the indicators.

Value [S]	Significance Category	Description
0	Not applicable	X
1	Insignificant	The subject is of little importance to the healthcare facility and there is a marginal tendency for evaluation.
2	Reduced significance	Failure to comply with this requirement could adversely affect the activity of the healthcare facility.
3	Significant	Failure to comply with the requirement could compromise the activity of the healthcare facility. It is essential to meet the requirement for healthcare.
4	Very significant	Failure to meet this requirement could jeopardize the successful provision of healthcare. Fulfilling the requirement is essential for the successful delivery of healthcare.
5	High significance	Failure to comply with the requirement may even compromise the existence of the healthcare facility.

**Table 3:** The indicator P.A.2.1—Healthcare services accessibility.

Indicator	P.A.2.1—Healthcare Services Accessibility
Indicator description	Healthcare services adapted to the specifics and requirements of the population.
Evaluation questions	Is the specificity of the treated patients identified? Are the needs of the population regarding healthcare services identi-fied? Does the design of healthcare services take into account the specifics of the patients and the requirements of the population? Do the healthcare services provided meet the specific needs of patients and the needs of the population?

**Table 4:** Indicator evaluation grid P.A.2.1—Healthcare services accessibility.

Value [G]	Completion Degree	Description
0	Not applicable	X
1	Weak	The specifics of the treated patients are identified.
2	Satisfactorily	The current and special needs of the treated patients are iden-tified.
3	Good	The explicit requirements of the population regarding healthcare services are collected.
4	Very good	In designing healthcare services, the specifics of the patients and the requirements of the population are taken into account.
5	Excellent	The healthcare services provided meet the specific needs of patients and the needs of the population.

**Table 5:** The indicator P.A.2.2—Medical care services for disadvantaged groups.

Indicator	P.A.2.2—Medical Care Services for Disadvantaged Groups
Indicator description	Healthcare services for vulnerable/disadvantaged groups.
Evaluation questions	Does the planned care provide care for disadvantaged groups? Are there specific services for disadvantaged people? If yes: what are they? Total number of healthcare services for vulnera-ble groups. If not: why?

**Table 6:** Indicator evaluation grid P.A.2.2—Medical care services for disadvantaged groups.

Value [G]	Completion Degree	Description
0	Not applicable	X
1	Weak	Healthcare planning does not include care services for disad-vantaged groups, and this is well-motivated.
2	Satisfactorily	Care planning for disadvantaged groups is provided in healthcare planning.
3	Good	TThere are specific healthcare services for disadvantaged peo-ple.
4	Very good	The total number of healthcare services for vulnerable groups covers the identified requirements.
5	Excellent	There is a continuing concern for the identification of new care services for disadvantaged groups, and their number is grow-ing since the previous assessment.

**Table 7:** The indicator P.B.2—Interventions with positive effects on patient satisfaction.

Indicator	P.B.2—Interventions with Positive Effects on Patient Satisfaction
Indicator description	Employment of interventions that indicate positive effects on patient satisfaction: _the art of care; _technical quality of care; _evaluation of total satisfaction.
Evaluation questions	Are patient-centered interventions applied to their satisfaction? Does the art of patient-centered care have positive effects on patient satisfaction? Is the technical quality of care improved?

**Table 8:** Indicator evaluation grid P.B.2—Interventions with positive effects on patient satisfaction.

Value [G]	Completion Degree	Description
0	Not applicable	X
1	Weak	The healthcare provided is patient centered.
2	Satisfactorily	Patient satisfaction with healthcare is assessed.
3	Good	Plans are being developed to improve healthcare services.
4	Very good	Patient satisfaction increases with patient-centered healthcare.
5	Excellent	The technical quality of healthcare is improved as a result of the use of new medical technologies.

**Table 9:** The indicator I.A.2—Specific medical approaches.

Indicator	I.A.2—Specific Medical Approaches
Indicator description	Appropriate and relevant medical approach to the patient. Adaptation to specific patient constraints and situations, provision of a common place to wait/prepare/change clothes, program adaptation to patient availability, provision of relevant medical information.
Evaluation questions	How are patients’ specific constraints and situations assessed? Are the conditions for medical services provision adapted to the identified specific requirements? Is there a common place for patients to wait/prepare/change clothes? Is the medical service delivery schedule tailored to patient availability? Are patients provided with relevant medical information?

**Table 10:** Indicator evaluation grid I.A.2—Specific medical approaches.

Value [G]	Completion Degree	Description
0	Not applicable	X
1	Weak	Patients’ specific constraints and situations are assessed indi-vidually and in groups.
2	Satisfactorily	The medical services provided to patients are tailored to the identified specific requirements.
3	Good	Patients have a common place to wait/prepare/change clothes.
4	Very good	The medical service provision program is adapted to the availability of patients.
5	Excellent	Patients are provided with relevant medical information tai-lored to their specific constraints and situations.

**Table 11:** The indicatorI.B.2—Fair transfer interventions.

Indicator	I.B.2—Fair Transfer Interventions
Indicator description	Transfer interventions must be visible, clear and fair. Transfers and associated interventions must be carried out with respect for the human dignity, social, national, racial and ethnic origin of pa-tients and the confidentiality of personal data.
Evaluation questions	Are the transfer interventions visible, clear and fair? Are the transfers and associated interventions made while respecting the human dignity, social, national, racial and ethnic origin of patients? Is the confidentiality of personal data maintained during transfers?

**Table 12:** Indicator evaluation grid I.B.2—Fair transfer interventions.

Value [G]	Completion Degree	Description
0	Not applicable	X
1	Weak	Legislation on the approval of inter clinical transfer protocols is available and enforced.
2	Satisfactorily	The medical staff informs the patient or their relatives about the risks and possible benefits of the transfer. If acceptance is not obtained, this situation and the reasons for the refusal are recorded in the patient’s file.
3	Good	The main purpose of the transfer is to ensure optimal healthcare for the patient, and the transfer interventions are visible, clear and fair. The confidentiality of personal data is maintained during transfers.
4	Very good	Transfers and associated interventions are carried out re-specting the human dignity, social, national, racial and ethnic origin of patients.
5	Excellent	The dignity of the patient in critical condition/terminal phase and their spiritual/cultural beliefs, previous decisions related to this event are taken into account.

**Table 13:** The indicator E.A.2.—Evaluation of current medical practices.

Indicator	E.A.2.—Evaluation of Current Medical Practices
Indicator description	Assessments by local opinion leaders on current medical practices and identification of interventions to be improved.
Evaluation questions	Do local opinion leaders conduct assessments of current medical practices? Are there identified interventions which need improvement?

**Table 14:** Indicator evaluation grid E.A.2.—Evaluation of current medical practices.

Value [G]	Completion Degree	Description
0	Not applicable	X
1	Weak	There are local opinion leaders who have professional influ-ences on the health professional community.
2	Satisfactorily	Local opinion leaders analyze current medical practices.
3	Good	Local opinion leaders conduct assessments of current medical practices and provide feedback.
4	Very good	Necessary interventions are identified in order to improve medical services.
5	Excellent	Medical services are being improved as a result of the imple-mentation of interventions proposed by local opinion leaders.

**Table 15:** E.B.2—Patient satisfaction degree.

Indicator	E.B.2—Patient Satisfaction Degree
Indicator description	The measure of patient satisfaction with the medical service received.
Evaluation questions	Is patient satisfaction with the medical service received measured? What is the evolution of patient satisfaction compared to the previous assessment? What are the proposed improvement measures to increase patient satisfaction?

**Table 16:** Indicator evaluation grid E.B.2—Patient satisfaction degree.

Value [G]	Completion Degree	Description
0	Not applicable	X
1	Weak	There are up-to-date patient satisfaction assessment ques-tionnaires.
2	Satisfactorily	Patient satisfaction assessment questionnaires are distributed periodically according to a procedure.
3	Good	The patient satisfaction degree is measured in terms of the received medical service.
4	Very good	The evolution of patient satisfaction compared to the previous assessment is evaluated.
5	Excellent	Improvements are being made in order to increase patient satisfaction.

**Table 17:** R.A.2—Freedom of expression assurance.

Indicator	R.A.2—Freedom of Expression Assurance
Indicator description	Record of the feedback collected (participants’ comments and sugges-tions) for continuous improvement in healthcare delivery.
Evaluation questions	How are the patients and medical staff comments and suggestions taken into account (formalized)? How are these registered?

**Table 18:** Indicator evaluation grid R.A.2—Freedom of expression assurance.

Value [G]	Completion Degree	Description
0	Not applicable	X
1	Weak	There is a system for collecting the feedback and suggestions from patients and healthcare professionals.
2	Satisfactorily	Observations and suggestions from patients and healthcare professionals are recorded and can be easily identified.
3	Good	The comments and suggestions collected from patients and medical staff are analyzed and improvement measures are formulated.
4	Very good	Improvement measures are applied, and resources are allo-cated.
5	Excellent	The implementation of improvement measures leads to the continuous improvement of healthcare provision.

**Table 19:** Self-assessment tool for human rights accountability.

No.	Symbol and Name of the Indicator	Significance Si	Completion Degree Gi	Quality Indicator ICi = Si × Gi
1	P.A.2.1 Healthcare services accessibility	5	4	20
2	P.A.2.2 Medical care services for disadvantaged groups	1	3	3
3	P.B.2 Interventions with positive effects on patient satisfaction	4	3	12
4	I.A.2 Specific medical approaches	2	5	10
5	I.B.2 Fair transfer interventions	3	5	15
6	E.A.2. Evaluation of current medical practices	4	4	16
7	E.B.2 Patient satisfaction degree	4	4	16
8	R.A.2 Freedom of expression assurance	2	3	6

## Data Availability

The data presented in this study are available on request from the corresponding author.
